# Maximum norm error estimates of fourth-order compact difference scheme for the nonlinear Schrödinger equation involving a quintic term

**DOI:** 10.1186/s13660-018-1775-y

**Published:** 2018-07-20

**Authors:** Hanqing Hu, Hanzhang Hu

**Affiliations:** School of Mathematics, Jiaying University, Meizhou, P.R. China

**Keywords:** Schrödinger equation involving a quintic term, Compact finite difference scheme, Conservation, Convergence, Unconditional stability, The max norm

## Abstract

A compact finite difference (CFD) scheme is presented for the nonlinear Schrödinger equation involving a quintic term. The two discrete conservative laws are obtained. The unconditional stability and convergence in maximum norm with order $O({\tau }^{2}+h^{4})$ are proved by using the energy method. A numerical experiment is presented to support our theoretical results.

## Introduction

The Schrödinger (NLS) equation is one of the most important equations of mathematical physics with applications in many fields [1–4] such as plasma physics, nonlinear optics, water waves, and bimolecular dynamics. There are many studies on numerical approaches, including finite difference [5–11], finite element [12–14], and polynomial approximation methods [15, 16], of the initial or initial-boundary value problems of the Schrödinger equations. We consider the initial-boundary value problem for the NLS equation involving a quintic term:

1.1 $$\begin{aligned} &i\frac{\partial u}{\partial t}+ \frac{\partial^{2} u}{\partial x^{2}}-\bigl( \vert u \vert ^{2}+ \vert u \vert ^{4}\bigr)u=f(x,t)u\quad (x_{l} < x < x _{r},0 < t \leq T), \end{aligned}$$

1.2 $$\begin{aligned} &u(x, 0)=u_{0}(x)\quad (x_{l} < x < x_{r}), \end{aligned}$$

1.3 $$\begin{aligned} &u(x_{l}, t)=u(x_{r}, t)=0\quad (0 < t \leq T), \end{aligned}$$

where $u(x, t)$ is a complex function, $f(x, t)$ is a real function, $u_{0}(x)$ is a prescribed smooth function, and $i^{2}=-1$.

Computing the inner product of equation (1.1) with *u* and $\frac{\partial u}{\partial t}$ and then taking the imaginary part and the real part, respectively, the two conservative laws are obtained as follows:

1.4 $$\begin{aligned} &Q(t) = \Vert u \Vert ^{2}_{L_{2}}=Q(0), \end{aligned}$$

1.5 $$\begin{aligned} &E(t) = \biggl\Vert \frac{\partial u}{\partial x} \biggr\Vert ^{2}_{L_{2}}+ \int^{x_{r}}_{x _{l}}\biggl(\frac{1}{2} \vert u \vert ^{4}+\frac{1}{2} \vert u \vert ^{6}\biggr)\, \mathrm{d}x=E(0)- \int^{t } _{0} \int^{x_{r}}_{x_{l}}f(x,t)\frac{\partial }{\partial t} \vert u \vert ^{2} \,\mathrm{d}x\,\mathrm{d}t, \end{aligned}$$

where $\|\cdot\|_{L_{2}}$ is the ${L_{2}}$ norm.

Zhang et al. found that the nonconservative schemes may easily show nonlinear blow-up when studying for NLS equation, so they presented a conservative difference scheme in [11]. Moreover, extensive mathematical and numerical studies have been carried out for the NLS equations in the literature [17–28]. Zhang presented a difference scheme for the NLS equation involving a quintic term [27], and it was proved with order $O({\tau }^{2}+h^{2})$. Then, in [28] Wang proposed a new difference scheme for NLS equation involving a quintic term and showed that convergence rates of the present scheme were of order $O({\tau }^{2}+h ^{4})$. Wang presented a compact finite difference scheme for the NLS equation in [22], which provided a new thinking on the theoretical proving of a compact difference scheme. There are lots of literature works concerning the Schrödinger equations using different treatments, but, to the best of our knowledge, there are few results of unconditional maximum norm convergence of compact difference scheme for NLS equations involving a quintic term. Thus, the purpose of this paper is to prove maximum norm error estimates of a fourth-order compact difference scheme for the NLS equation involving a quintic term.

The remainder of this paper is organized as follows. A fourth-order compact difference scheme is proposed in Sect. 2. The discrete conservation laws of the difference scheme are discussed in Sect. 3. In Sect. 4, the convergence and stability for the compact difference scheme are proved. In the last section, numerical results will be discussed.

## Some notations and compact finite difference scheme

For simplicity of exposition, some notations are firstly introduced. Thus, the following notations for difference operators are used:

$$\begin{aligned} &\delta_{t}u^{n}_{j}=\frac{u^{n}_{j}-u^{n-1}_{j}}{\tau }, \qquad \delta _{x}u^{n}_{j}=\frac{u^{n}_{j+1}-u^{n}_{j}}{h}, \qquad \delta_{\bar{x}}u^{n} _{j}=\frac{u^{n}_{j}-u^{n}_{j-1}}{h},\qquad u^{n+\frac{1}{2}}_{j}=\frac{u ^{n+1}_{j}+u^{n}_{j}}{2}, \\ &\delta^{2}_{x}u^{n}_{j}= \delta_{x}\delta_{\bar{x}}u^{n}_{j}= \frac{u ^{n}_{j-1}-2u^{n}_{j}+u^{n}_{j+1}}{h^{2}},\qquad A_{h}u^{n}_{j}=u^{n}_{j}+ \frac{h ^{2}}{12}\delta^{2}_{x}u^{n}_{j}= \frac{1}{12}\bigl(u^{n}_{j-1}+10u^{n}_{j}+u ^{n}_{j+1}\bigr), \end{aligned}$$

where $h=\frac{x_{r}-x_{l}}{J}$ and $\tau =\frac{T}{N}$ are step sizes of space and time, respectively, and *J*, *N* are two positive integers.

For any $\pmb{u}, \pmb{v}\in V_{h}=\{\pmb{v}|\pmb{v}=(v_{0},v _{1},\ldots,v_{J}),v_{0}=v_{J}=0\}$, the inner product is defined as

$$ (\pmb{u},\pmb{v})=h\sum^{J-1}_{j=1}u_{j} \bar{v}_{j}. $$

The discrete norms of *u* are defined as

$$\begin{aligned} \Vert \pmb{u} \Vert ^{p}_{p}=h\sum ^{J-1}_{j=1} \vert u_{j} \vert ^{p},\qquad \Vert \delta_{x}\pmb{u} \Vert ^{2}=h\sum^{J-1}_{j=0} \vert \delta_{x}u_{j} \vert ^{2},\qquad \Vert \pmb{u} \Vert _{ \infty }=\max_{1\le j \le J-1} \vert u_{j} \vert . \end{aligned}$$

For simplicity, we define $\{U^{n}_{j}\}$ as the exact solution and $\{u^{n}_{j}\}$ as the numerical one. Let *C* denote a positive constant independent of discretization parameters, but it may have different values at different occurrences. For the exact solution of the initial-boundary value problem (1.1)–(1.3), we assume that

2.1 $$ \max \bigl\{ \bigl\Vert U^{n} \bigr\Vert , \bigl\Vert \delta_{x}U^{n} \bigr\Vert , \bigl\Vert U^{n} \bigr\Vert _{\infty }\bigr\} \leq C. $$

Now, we present the following compact finite difference scheme for problem (1.1)–(1.3):

2.2 $$\begin{aligned} &iA_{h}\delta_{t}u^{n}_{j}+ \frac{1}{2}\delta^{2}_{x}\bigl(u^{n+1}_{j}+u ^{n}_{j}\bigr) -\frac{1}{4}A_{h}\bigl[ \bigl( \bigl\vert u^{n+1}_{j} \bigr\vert ^{2}+ \bigl\vert u^{n}_{j} \bigr\vert ^{2}\bigr) \bigl(u^{n+1} _{j}+u^{n}_{j}\bigr)\bigr] \\ &\quad {}-\frac{1}{6}A_{h}\bigl[\bigl( \bigl\vert u^{n+1}_{j} \bigr\vert ^{4}+ \bigl\vert u^{n+1}_{j} \bigr\vert ^{2} \bigl\vert u^{n}_{j} \bigr\vert ^{2}+ \bigl\vert u ^{n}_{j} \bigr\vert ^{4}\bigr) \bigl(u^{n+1}_{j}+u^{n}_{j}\bigr) \bigr]=A_{h}\biggl[f^{n+\frac{1}{2}}\frac{u ^{n+1}_{j}+u^{n}_{j}}{2}\biggr] \\ &\quad (j=1, 2, \ldots, J-1, n=1, 2, \ldots, N-1), \end{aligned}$$

2.3 $$\begin{aligned} &u^{n}_{0}=u^{n}_{J}=0\quad (n=1, 2, \ldots, N), \end{aligned}$$

2.4 $$\begin{aligned} &u^{0}_{J}=u_{0}(x_{j})\quad (j=1, 2, \ldots, J). \end{aligned}$$

Let

$$\begin{aligned} &\pmb{u}^{n}=\bigl(u^{n}_{0},u^{n}_{1}, \ldots,u^{n}_{J-1}\bigr)^{T}, \\ & \bigl\vert \pmb{u}^{n+1} \bigr\vert ^{2}+ \bigl\vert \pmb{u}^{n} \bigr\vert ^{2}=\pmb{\operatorname{diag}}\bigl( \bigl\vert u^{n+1}_{0} \bigr\vert ^{2}+ \bigl\vert u ^{n}_{0} \bigr\vert ^{2},\ldots, \bigl\vert u^{n+1}_{J-1} \bigr\vert ^{2}+ \bigl\vert u^{n}_{J-1} \bigr\vert ^{2}\bigr). \end{aligned}$$

(2.2) can be rewritten as

$$\begin{aligned} &i\pmb{M}\delta_{t}\pmb{u}^{n}+\frac{1}{2} \delta^{2}_{x}\bigl(\pmb{u}^{n+1}+\pmb{u} ^{n}\bigr) -\frac{1}{4}\pmb{M}\bigl( \bigl\vert \pmb{u}^{n+1} \bigr\vert ^{2}+ \bigl\vert \pmb{u}^{n} \bigr\vert ^{2}\bigr) \bigl(\pmb{u} ^{n+1}+\pmb{u}^{n}\bigr) \\ &\quad -\frac{1}{6}\pmb{M}\bigl( \bigl\vert \pmb{u}^{n+1} \bigr\vert ^{4}+ \bigl\vert \pmb{u}^{n} \bigr\vert ^{2} \bigl\vert \pmb{u} ^{n+1} \bigr\vert ^{2}+ \bigl\vert \pmb{u}^{n} \bigr\vert ^{4}\bigr) \bigl( \pmb{u}^{n+1}+\pmb{u}^{n}\bigr) =\pmb{M}f ^{n+\frac{1}{2}} \frac{\pmb{u}^{n+1}+\pmb{u}^{n}}{2}, \\ &\quad n=1, 2, \ldots, N-1, \end{aligned}$$

where the matrix *M* is defined by

$$ \pmb{M}=\frac{1}{12} \left ( \begin{matrix} 10 &1 &0 &\cdots &0 \\ 1 &10 &1 &\cdots &0 \\ &\ddots &\ddots &\ddots & \\ 0 &\cdots &0 &1 &10 \end{matrix} \right ) _{(J-1)\times (J-1)}. $$

$\pmb{M}$ is a tridiagonal symmetric matrix, and there is a symmetric positive definite matrix $\pmb{H}$ such that $\pmb{H}=\pmb{M}^{-1}$. Thus, the compact finite difference scheme (2.2)–(2.4) can be rewritten as the following matrix equation:

2.5 $$\begin{aligned} &i\delta_{t}\pmb{u}^{n}+ \frac{1}{2}\pmb{H}\delta^{2}_{x}\bigl( \pmb{u}^{n+1}+\pmb{u} ^{n}\bigr) -\frac{1}{4}\bigl( \bigl\vert \pmb{u}^{n+1} \bigr\vert ^{2}+ \bigl\vert \pmb{u}^{n} \bigr\vert ^{2}\bigr) \bigl( \pmb{u}^{n+1}+\pmb{u} ^{n}\bigr) \\ &\quad {}-\frac{1}{6}\bigl( \bigl\vert \pmb{u}^{n+1} \bigr\vert ^{4}+ \bigl\vert \pmb{u}^{n+1} \bigr\vert ^{2} \bigl\vert \pmb{u}^{n} \bigr\vert ^{2}+ \bigl\vert \pmb{u} ^{n} \bigr\vert ^{4}\bigr) \bigl( \pmb{u}^{n+1}+\pmb{u}^{n}\bigr) =\pmb{f}^{n+\frac{1}{2}} \frac{\pmb{u} ^{n+1}+\pmb{u}^{n}}{2} \\ &\quad (n=1, 2, \ldots, N-1), \end{aligned}$$

2.6 $$\begin{aligned} &u^{n}_{0}=u^{n}_{J}=0\quad (n=1, 2, \ldots, N), \end{aligned}$$

2.7 $$\begin{aligned} &u^{0}_{J}=u_{0}(x_{j})\quad (j=0,1, 2, \ldots, J). \end{aligned}$$

## Some useful lemmas and discrete conservation laws

### Lemma 3.1

([29])

*For any two mesh functions* $\pmb{u},\pmb{v}\in V_{h}$, *there is*

$$ h\sum^{J-1}_{j=1}\bigl(\delta^{2}_{x} u_{j}\bigr)\bar{v}_{j}=-h\sum^{J-1}_{j=1}( \delta_{x} u_{j}) (\delta_{x} \bar{v}_{j}). $$

### Lemma 3.2

([22])

*For any real symmetric positive definite matrices* $\pmb{H}$, *we have*

$$ \operatorname{Re}\bigl(\pmb{H}\delta^{2}_{x}\bigl(\pmb{u}^{n+1}+ \pmb{u}^{n}\bigr),\pmb{u}^{n+1}-\pmb{u} ^{n}\bigr)=- \bigl( \bigl\Vert \pmb{R}\delta_{x}\pmb{u}^{n+1} \bigr\Vert ^{2}- \bigl\Vert \pmb{R}\delta_{x}\pmb{u} ^{n} \bigr\Vert ^{2}\bigr), $$

*where* $\pmb{R}$ *is obtained by the Cholesky decomposition for* $\pmb{H}$, *denoted as* $\pmb{R}= \operatorname{chol}(\pmb{H})$.

### Theorem 3.1

*The difference scheme* (2.2)*–*(2.4) *is conservative in the sense*

3.1 $$\begin{aligned} &Q^{n} = \bigl\Vert \pmb{u}^{n} \bigr\Vert =Q^{n-1}=\cdots =Q^{0}, \end{aligned}$$

3.2 $$\begin{aligned} &E^{n} = \bigl\Vert \pmb{R}\delta_{x} \pmb{u}^{n} \bigr\Vert ^{2}+\frac{1}{2} \bigl\Vert \pmb{u}^{n} \bigr\Vert ^{4}_{4}+\frac{1}{3} \bigl\Vert \pmb{u}^{n} \bigr\Vert ^{6}_{6}+h \sum^{J-1}_{j=1}f^{n- \frac{1}{2}}_{j} \bigl\vert u^{n}_{j} \bigr\vert ^{2} \\ &\hphantom{E^{n}} =E^{n-1}+h\sum^{J-1}_{j=1} \bigl(f^{n- \frac{1}{2}}_{j}-f^{n-\frac{3}{2}}_{j}\bigr) \bigl\vert u^{n}_{j} \bigr\vert ^{2} \\ &\hphantom{E^{n}}=E^{0}+\sum^{n}_{l=1} \sum^{J-1}_{j=1}\bigl(f^{l-\frac{1}{2}}_{j}-f^{l- \frac{3}{2}}_{j} \bigr) \bigl\vert u^{n}_{j} \bigr\vert ^{2}h, \end{aligned}$$

*for* $n=1, 2, \ldots, N$, *where* $Q^{n}$ *is discrete mass*, $E^{n}$ *is discrete energy*.

### Proof

Computing the inner product of (2.2) with $\pmb{u}^{n+1}+\pmb{u} ^{n}$ and then taking the imaginary part, we obtain

$$ I_{1}+I_{2}-I_{3}-I_{4}=I_{5}, $$

where

$$\begin{aligned} &I_{1} =\operatorname{Im}\bigl(i\delta_{t}\pmb{u}^{n}, \pmb{u}^{n+1}+\pmb{u}^{n}\bigr)=\operatorname{Re}\bigl(\delta _{t} \pmb{u}^{n},\pmb{u}^{n+1}+\pmb{u}^{n}\bigr)= \frac{1}{\tau }\bigl( \bigl\Vert \pmb{u} ^{n+1} \bigr\Vert ^{2}- \bigl\Vert \pmb{u}^{n} \bigr\Vert ^{2} \bigr), \\ &I_{2} =\frac{1}{2}\operatorname{Im}\bigl(\pmb{H}\delta^{2}_{x} \bigl(\pmb{u}^{n+1}+\pmb{u}^{n}\bigr),\pmb{u} ^{n+1}+ \pmb{u}^{n}\bigr)=-2\operatorname{Im}\bigl(\pmb{R}\delta_{x} \pmb{u}^{n+\frac{1}{2}},\pmb{R} \delta_{x}\pmb{u}^{n+\frac{1}{2}}\bigr)=0, \\ &I_{3} =\frac{1}{4}\operatorname{Im}\bigl(\bigl( \bigl\vert \pmb{u}^{n+1} \bigr\vert ^{2}+ \bigl\vert \pmb{u}^{n} \bigr\vert ^{2}\bigr) \bigl(\pmb{u} ^{n+1}+\pmb{u}^{n}\bigr),\pmb{u}^{n+1}+ \pmb{u}^{n}\bigr)=0, \\ &I_{4} =\frac{1}{6}\operatorname{Im}\bigl(\bigl( \bigl\vert \pmb{u}^{n+1} \bigr\vert ^{4}+ \bigl\vert \pmb{u}^{n+1} \bigr\vert ^{2} \bigl\vert \pmb{u} ^{n} \bigr\vert ^{2}+ \bigl\vert \pmb{u}^{n} \bigr\vert ^{4}\bigr) \bigl(\pmb{u}^{n+1}+ \pmb{u}^{n}\bigr),\pmb{u}^{n+1}+\pmb{u} ^{n}\bigr)=0, \\ &I_{5} =\frac{1}{2}\operatorname{Im}\bigl(\pmb{f}^{n+\frac{1}{2}}\bigl( \pmb{u}^{n+1}+\pmb{u} ^{n}\bigr),\pmb{u}^{n+1}+ \pmb{u}^{n}\bigr)=0. \end{aligned}$$

We can obtain

$$ \bigl\Vert \pmb{u}^{n+1} \bigr\Vert ^{2}- \bigl\Vert \pmb{u}^{n} \bigr\Vert ^{2}=0. $$

Then we have

$$ Q^{n}= \bigl\Vert \pmb{u}^{n} \bigr\Vert =Q^{n-1}=\cdots =Q^{0}. $$

Computing the inner product of (2.2) with $\pmb{u}^{n+1}-\pmb{u} ^{n}$, and then taking the real part, we get

$$ I_{6}+I_{7}-I_{8}-I_{9}=I_{10}, $$

where

$$\begin{aligned} &I_{6} =\operatorname{Re}\tau \bigl(i\delta_{t}\pmb{u}^{n}, \delta_{t}\pmb{u}^{n}\bigr)=0, \\ &I_{7} =\frac{1}{2}\operatorname{Re}\bigl(\pmb{H}\delta^{2}_{x} \bigl(\pmb{u}^{n+1}+\pmb{u}^{n}\bigr),\pmb{u} ^{n+1}- \pmb{u}^{n}\bigr)=-\frac{1}{2}\bigl( \bigl\Vert \pmb{R} \delta_{x}\pmb{u}^{n+1} \bigr\Vert ^{2}- \bigl\Vert \pmb{R}\delta_{x}\pmb{u}^{n} \bigr\Vert ^{2}\bigr), \\ &I_{8} =\frac{1}{4}\operatorname{Re}\bigl(\bigl( \bigl\vert \pmb{u}^{n+1} \bigr\vert ^{2}+ \bigl\vert \pmb{u}^{n} \bigr\vert ^{2}\bigr) \bigl(\pmb{u} ^{n+1}+\pmb{u}^{n}\bigr),\pmb{u}^{n+1}- \pmb{u}^{n}\bigr)=\frac{1}{4}\bigl( \bigl\Vert \pmb{u} ^{n+1} \bigr\Vert ^{4}_{4}- \bigl\Vert \pmb{u}^{n} \bigr\Vert ^{4}_{4}\bigr), \\ &I_{9} =\frac{1}{6}\operatorname{Re}\bigl(\bigl( \bigl\vert \pmb{u}^{n+1} \bigr\vert ^{4}+ \bigl\vert \pmb{u}^{n+1} \bigr\vert ^{2} \bigl\vert \pmb{u} ^{n} \bigr\vert ^{2}+ \bigl\vert \pmb{u}^{n} \bigr\vert ^{4}\bigr) \bigl(\pmb{u}^{n+1}+ \pmb{u}^{n}\bigr),\pmb{u}^{n+1}-\pmb{u} ^{n}\bigr) \\ &\hphantom{I_{9}}= \frac{1}{6}\bigl( \bigl\Vert \pmb{u}^{n+1} \bigr\Vert ^{6}_{6}- \bigl\Vert \pmb{u}^{n} \bigr\Vert ^{6}_{6}\bigr), \\ &I_{10} =\frac{1}{2}\operatorname{Re}\bigl(\pmb{f}^{n+\frac{1}{2}}\bigl( \pmb{u}^{n+1}+\pmb{u} ^{n}\bigr),\pmb{u}^{n+1}- \pmb{u}^{n}\bigr)=\frac{h}{2}\sum^{J-1}_{j=1}f^{n+ \frac{1}{2}} \bigl( \bigl\vert u^{n+1}_{j} \bigr\vert ^{2}- \bigl\vert u^{n}_{j} \bigr\vert ^{2}\bigr). \end{aligned}$$

Let

$$ E^{n}= \bigl\Vert \pmb{R}\delta_{x}\pmb{u}^{n} \bigr\Vert ^{2}+\frac{1}{2} \bigl\Vert \pmb{u}^{n} \bigr\Vert ^{4}_{4}+\frac{1}{3} \bigl\Vert \pmb{u}^{n} \bigr\Vert ^{6}_{6}+h\sum ^{J-1}_{j=1}f^{n- \frac{1}{2}}_{j} \bigl\vert u^{n}_{j} \bigr\vert ^{2}. $$

We can obtain

$$ E^{n}=E^{n-1}+h\sum^{J-1}_{j=1} \bigl(f^{n-\frac{1}{2}}_{j}-f^{n- \frac{3}{2}}_{j}\bigr) \bigl\vert u^{n}_{j} \bigr\vert ^{2}. $$

Summing up for n, we have

$$ E^{n}=E^{0}+\sum^{n}_{l=1} \sum^{J-1}_{j=1}\bigl(f^{l-\frac{1}{2}}_{j}-f ^{l-\frac{3}{2}}_{j}\bigr) \bigl\vert u^{n}_{j} \bigr\vert ^{2}h. $$

 □

## Numerical analysis

To obtain the error estimate in the maximum norm, we need the following lemmas.

### Lemma 4.1

(Discrete Sobolev’s inequality [30])

*Suppose that* ${u_{j}}$ *is mesh functions*. *Given* $\varepsilon \geq 0 $, *there exists a constant C dependent on* *ε* *such that*

$$ \Vert u \Vert _{\infty } \leq \varepsilon \Vert \delta_{x}u \Vert +C \Vert u \Vert . $$

### Lemma 4.2

(Gronwall’s inequality [30])

*Suppose that the nonnegative mesh function* $\{ u^{n}|n=0, 1, 2, \ldots, N, N\tau =T\}$ *satisfies the inequality*

$$ u^{n}\leq A+\tau \sum^{n}_{l=1}B_{k}u^{k}, $$

*where A and* $B_{k}$ ($k=1, 2, \ldots, N, N\tau =T$) *satisfying the inequality are nonnegative constants*. *Then*, *for any* $0\leq n \leq N$, *there is*

$$ \bigl\Vert u^{n} \bigr\Vert _{\infty }\leq Ae^{2\tau \sum^{N}_{k=1}B_{k}}, $$

*where* *τ* *is sufficiently small such that* $\tau ({\max }_{k=1, 2, \ldots, N} B_{k})\leq \frac{1}{2}$.

### Lemma 4.3

([22])

*For any real symmetric positive definite matrices* $\pmb{H}$, *there exist two positive numbers* $C_{*}$ *and* $C^{*}$ *such that*

$$ C_{*} \bigl\Vert \pmb{u}^{n} \bigr\Vert ^{2} \leq \bigl(\pmb{H}\pmb{u}^{n},\pmb{u}^{n}\bigr)\leq C ^{*} \bigl\Vert \pmb{u}^{n} \bigr\Vert ^{2}. $$

### Theorem 4.1

*Suppose that* $|f(x,t)|\leq M_{1}$, $|f_{t}(x,t)|\leq M_{2}$, $u_{0} \in H^{1}_{0} $, *then*, *for any* *n* ($0\leq n\tau \leq T$), *the following estimates hold*:

$$ \bigl\Vert \pmb{u}^{n} \bigr\Vert \leq C,\qquad \bigl\Vert \pmb{u}^{n} \bigr\Vert _{\infty }\leq C. $$

### Proof

From (3.1), we have

4.1 $$ \bigl\Vert \pmb{u}^{n} \bigr\Vert \leq C. $$

From (3.2), we obtain

$$ \bigl\Vert \pmb{R}\delta_{x}\pmb{u}^{n} \bigr\Vert ^{2}+\frac{1}{2} \bigl\Vert \pmb{u}^{n} \bigr\Vert ^{4} _{4}+\frac{1}{3} \bigl\Vert \pmb{u}^{n} \bigr\Vert ^{6}_{6}+h\sum ^{J-1}_{j=1}f^{n- \frac{1}{2}}_{j} \bigl\vert u^{n}_{j} \bigr\vert ^{2} =E^{0}+\sum^{n}_{l=1}\sum ^{J-1}_{j=1}\bigl(f ^{l-\frac{1}{2}}_{j}-f^{l-\frac{3}{2}}_{j} \bigr) \bigl\vert u^{n}_{j} \bigr\vert ^{2}h, $$

thus, we have

$$ \bigl\Vert \pmb{R}\delta_{x}\pmb{u}^{n} \bigr\Vert ^{2} \leq E^{0}+\sum^{n}_{l=1} \sum^{J-1}_{j=1}\bigl(f^{l-\frac{1}{2}}_{j}-f^{l-\frac{3}{2}}_{j} \bigr) \bigl\vert u^{n}_{j} \bigr\vert ^{2}h-h \sum^{J-1}_{j=1}f^{n-\frac{1}{2}}_{j} \bigl\vert u^{n}_{j} \bigr\vert ^{2}. $$

On the one hand, from (4.1), we have

$$\begin{aligned} \bigl\Vert \pmb{R}\delta_{x}\pmb{u}^{n} \bigr\Vert ^{2} &\leq E^{0}+\sum^{n}_{l=1} \sum^{J-1}_{j=1}\bigl(f^{l-\frac{1}{2}}_{j}-f^{l-\frac{3}{2}}_{j} \bigr) \bigl\vert u^{n}_{j} \bigr\vert ^{2}h-h \sum^{J-1}_{j=1}f^{n-\frac{1}{2}}_{j} \bigl\vert u^{n}_{j} \bigr\vert ^{2} \\ &\leq \bigl\vert E^{0} \bigr\vert +M_{1}h\sum ^{J-1}_{j=1} \bigl\vert u^{n}_{j} \bigr\vert ^{2}+h\sum^{n}_{l=1} \sum^{J-1}_{j=1} \biggl\vert \biggl( \frac{\partial f}{\partial t}\biggr)_{(j,l+\theta)} \biggr\vert \cdot \bigl\vert u^{l}_{j} \bigr\vert ^{2} \\ & \leq C. \end{aligned}$$

On the other hand, from Lemma 4.3, we have

$$ \bigl\Vert \pmb{R}\delta_{x}\pmb{u}^{n} \bigr\Vert ^{2}=\bigl(\pmb{H}\delta_{x}\pmb{u}^{n}, \delta_{x}\pmb{u}^{n}\bigr)\geq C_{*} \bigl\Vert \delta_{x}\pmb{u}^{n} \bigr\Vert ^{2}. $$

Then we see that

4.2 $$ \bigl\Vert \delta_{x}\pmb{u}^{n} \bigr\Vert \leq C. $$

From (4.1)–(4.2) and Lemma 4.1, we obtain

4.3 $$ \bigl\Vert \pmb{u}^{n} \bigr\Vert _{\infty }\leq C. $$

 □

Suppose that the truncation error

$$ \pmb{r}^{n}=\bigl(r^{n}_{0},r^{n}_{1}, \ldots,r^{n}_{J-1}\bigr)^{T}\in V_{h}, $$

then we have

4.4 $$\begin{aligned} \pmb{r}^{n}={} &i \delta_{t}\pmb{U}^{n}+\frac{1}{2}\pmb{H} \delta^{2}_{x}\bigl(\pmb{U} ^{n+1}+ \pmb{U}^{n}\bigr) -\frac{1}{4}\bigl( \bigl\vert \pmb{U}^{n+1} \bigr\vert ^{2}+ \bigl\vert \pmb{U}^{n} \bigr\vert ^{2}\bigr) \bigl(\pmb{U} ^{n+1}+\pmb{U}^{n}\bigr) \\ &{}-\frac{1}{6}\bigl( \bigl\vert \pmb{U}^{n+1} \bigr\vert ^{4}+ \bigl\vert \pmb{U}^{n} \bigr\vert ^{2} \bigl\vert \pmb{U}^{n+1} \bigr\vert ^{2}+ \bigl\vert \pmb{U} ^{n} \bigr\vert ^{4}\bigr) \bigl( \pmb{U}^{n+1}+\pmb{U}^{n}\bigr) -\pmb{f}^{n+\frac{1}{2}} \frac{\pmb{U} ^{n+1}+\pmb{U}^{n}}{2}. \end{aligned}$$

According to Taylor’s expansion, the following can be easily obtained.

### Lemma 4.4

*Suppose that* $u_{0}(x)\in H^{1}_{0}$, $u(x,t)\in C^{6,3}$, *then we have*

4.5 $$\begin{aligned} & \bigl\vert r^{n}_{j} \bigr\vert \leq O \bigl(h^{4}+\tau^{2}\bigr), \end{aligned}$$

4.6 $$\begin{aligned} & \bigl\vert \delta_{t} r^{n}_{j} \bigr\vert \leq O\bigl(h^{4}+\tau^{2}\bigr). \end{aligned}$$

### Lemma 4.5

[[22]] *For* $u=\{u^{0},u^{1},\ldots,u^{n},u^{n+1}\}$ *and* $g=\{g^{0},g ^{1},\ldots,g^{n-1},g^{n}\}$, *we have*

4.7 $$ \Biggl\vert 2\tau \sum^{n}_{l=0}g^{l} \delta_{t}u^{l} \Biggr\vert \leq \bigl\vert u^{0} \bigr\vert ^{2}+\tau \sum ^{n}_{l=1} \bigl\vert u^{l} \bigr\vert ^{2}+ \bigl\vert u^{n+1} \bigr\vert ^{2}+ \bigl\vert g^{0} \bigr\vert ^{2}+\tau \sum ^{n-1}_{l=0} \bigl\vert \delta_{t}g^{l} \bigr\vert ^{2}+ \bigl\vert g^{n} \bigr\vert ^{2}. $$

### Theorem 4.2

*Suppose that the conditions of Theorem* 4.1 *and Lemma* 4.4 *are satisfied*, *then the numerical solution of scheme* (2.2)*–*(2.4) *converges to the solution of problem* (1.1)*–*(1.3) *with order* $O(h^{4}+\tau^{2})$ *in the discrete* $\|\cdot \|_{\infty }$ *norm*.

### Proof

Let

$$ \pmb{e}^{n}=\pmb{U}^{n}-\pmb{u}^{n}. $$

Subtracting (2.5) from (4.4), we obtain

4.8 $$\begin{aligned} \begin{aligned} \pmb{r}^{n}=i \delta_{t}\pmb{e}^{n}+\frac{1}{2}\pmb{H} \delta^{2}_{x}\bigl(\pmb{e} ^{n+1}+ \pmb{e}^{n}\bigr) -\frac{1}{4}\pmb{F}^{n}- \frac{1}{6}\pmb{G}^{n} -\pmb{f} ^{n+\frac{1}{2}}\frac{\pmb{e}^{n+1}+\pmb{e}^{n}}{2}, \end{aligned} \end{aligned}$$

where

4.9 $$\begin{aligned} \begin{aligned}&\pmb{F}^{n}=\bigl( \bigl\vert \pmb{U}^{n+1} \bigr\vert ^{2}+ \bigl\vert \pmb{U}^{n} \bigr\vert ^{2} \bigr) \bigl(\pmb{U}^{n+1}+\pmb{U} ^{n}\bigr)-\bigl( \bigl\vert \pmb{u}^{n+1} \bigr\vert ^{2}+ \bigl\vert \pmb{u}^{n} \bigr\vert ^{2}\bigr) \bigl( \pmb{u}^{n+1}+\pmb{u} ^{n}\bigr), \\ &\pmb{G}^{n}=\bigl( \bigl\vert \pmb{U}^{n+1} \bigr\vert ^{4}+ \bigl\vert \pmb{U}^{n+1} \bigr\vert ^{2} \bigl\vert \pmb{U}^{n} \bigr\vert ^{2}+ \bigl\vert \pmb{U} ^{n} \bigr\vert ^{4}\bigr) \bigl( \pmb{U}^{n+1}+\pmb{U}^{n}\bigr) \\ &\hphantom{\pmb{G}^{n}=}{}-\bigl( \bigl\vert \pmb{u}^{n+1} \bigr\vert ^{4}+ \bigl\vert \pmb{u}^{n+1} \bigr\vert ^{2} \bigl\vert \pmb{u}^{n} \bigr\vert ^{2}+ \bigl\vert \pmb{u} ^{n} \bigr\vert ^{4}\bigr) \bigl(\pmb{u}^{n+1}+ \pmb{u}^{n}\bigr), \\ &\pmb{F}^{n}=\bigl( \bigl\vert \pmb{U}^{n+1} \bigr\vert ^{2}+ \bigl\vert \pmb{U}^{n} \bigr\vert ^{2}\bigr) \bigl( \pmb{U}^{n+1}+\pmb{U} ^{n}\bigr)-\bigl( \bigl\vert \pmb{u}^{n+1} \bigr\vert ^{2}+ \bigl\vert \pmb{u}^{n} \bigr\vert ^{2}\bigr) \bigl( \pmb{u}^{n+1}+\pmb{u} ^{n}\bigr) \\ &\hphantom{\pmb{F}^{n}}=\bigl[\bigl( \bigl\vert \pmb{U}^{n+1} \bigr\vert ^{2}+ \bigl\vert \pmb{U}^{n} \bigr\vert ^{2}\bigr)-\bigl( \bigl\vert \pmb{u}^{n+1} \bigr\vert ^{2}+ \bigl\vert \pmb{u} ^{n} \bigr\vert ^{2}\bigr)\bigr]\bigl(\pmb{U}^{n+1}+ \pmb{U}^{n}\bigr) \\ &\hphantom{\pmb{F}^{n}=}{}+\bigl( \bigl\vert \pmb{u}^{n+1} \bigr\vert ^{2}+ \bigl\vert \pmb{u} ^{n} \bigr\vert ^{2} \bigr) \bigl(\pmb{e}^{n+1}+\pmb{e}^{n}\bigr) \\ &\hphantom{\pmb{F}^{n}}=\bigl[\bigl(\pmb{U}^{n+1}\bar{\pmb{e}}^{n+1}+ \pmb{e}^{n+1}\bar{\pmb{U}}^{n+1}\bigr)+\bigl(\pmb{U} ^{n}\bar{\pmb{e}}^{n}+\pmb{e}^{n}\bar{\pmb{U}}^{n}\bigr)\bigr]\bigl(\pmb{U}^{n+1}+\pmb{U} ^{n}\bigr)+\bigl( \bigl\vert \pmb{u}^{n+1} \bigr\vert ^{2} \\ &\hphantom{\pmb{F}^{n}=}{}+ \bigl\vert \pmb{u}^{n} \bigr\vert ^{2} \bigr) \bigl(\pmb{e}^{n+1}+\pmb{e} ^{n}\bigr). \end{aligned} \end{aligned}$$

Noting that $F^{n}_{0}=F^{n}_{J}=0$, from (2.1), (4.9), and Theorem 4.1, we have

4.10 $$ \bigl\Vert \pmb{F}^{n} \bigr\Vert ^{2}\leq C\bigl( \bigl\Vert \pmb{e}^{n} \bigr\Vert ^{2}+ \bigl\Vert \pmb{e}^{n+1} \bigr\Vert ^{2}\bigr). $$

Similarly, we obtain

4.11 $$\begin{aligned}& \bigl\Vert \delta_{x}\pmb{F}^{n} \bigr\Vert ^{2}\leq C\bigl( \bigl\Vert \pmb{e}^{n} \bigr\Vert ^{2}+ \bigl\Vert \pmb{e}^{n+1} \bigr\Vert ^{2}+ \bigl\Vert \delta_{x} \pmb{e}^{n} \bigr\Vert ^{2}+ \bigl\Vert \delta_{x} \pmb{e}^{n+1} \bigr\Vert ^{2}\bigr), \end{aligned}$$

4.12 $$\begin{aligned}& \pmb{G}^{n}= \bigl( \bigl\vert \pmb{U}^{n+1} \bigr\vert ^{4}+ \bigl\vert \pmb{U}^{n+1} \bigr\vert ^{2} \bigl\vert \pmb{U}^{n} \bigr\vert ^{2}+ \bigl\vert \pmb{U} ^{n} \bigr\vert ^{4}\bigr) \bigl(\pmb{U}^{n+1}+ \pmb{U}^{n}\bigr) \\& \hphantom{\pmb{G}^{n}=}{}-\bigl( \bigl\vert \pmb{u}^{n+1} \bigr\vert ^{4}+ \bigl\vert \pmb{u} ^{n+1} \bigr\vert ^{2} \bigl\vert \pmb{u}^{n} \bigr\vert ^{2}+ \bigl\vert \pmb{u}^{n} \bigr\vert ^{4}\bigr) \bigl( \pmb{u}^{n+1}+\pmb{u} ^{n}\bigr) \\& \hphantom{\pmb{G}^{n}}= \bigl[\bigl( \bigl\vert \pmb{U}^{n+1} \bigr\vert ^{4}+ \bigl\vert \pmb{U}^{n+1} \bigr\vert ^{2} \bigl\vert \pmb{U}^{n} \bigr\vert ^{2}+ \bigl\vert \pmb{U} ^{n} \bigr\vert ^{4}\bigr)-\bigl( \bigl\vert \pmb{u}^{n+1} \bigr\vert ^{4}+ \bigl\vert \pmb{u}^{n+1} \bigr\vert ^{2} \bigl\vert \pmb{u}^{n} \bigr\vert ^{2}+ \bigl\vert \pmb{u} ^{n} \bigr\vert ^{4}\bigr)\bigr] \\& \hphantom{\pmb{G}^{n}=}{}\times \bigl(\pmb{U}^{n+1}+ \pmb{U}^{n}\bigr) \\& \hphantom{\pmb{G}^{n}=}{}+\bigl( \bigl\vert \pmb{u}^{n+1} \bigr\vert ^{4}+ \bigl\vert \pmb{u}^{n+1} \bigr\vert ^{2} \bigl\vert \pmb{u}^{n} \bigr\vert ^{2}+ \bigl\vert \pmb{u} ^{n} \bigr\vert ^{4}\bigr) \bigl(\pmb{e}^{n+1}+ \pmb{e}^{n}\bigr) \\& \hphantom{\pmb{G}^{n}}= \bigl[\bigl( \bigl\vert \pmb{U}^{n+1} \bigr\vert ^{2}+ \bigl\vert \pmb{U}^{n+1} \bigr\vert ^{2}\bigr) \bigl( \pmb{U}^{n+1}\bar{\pmb{e}} ^{n+1}+\pmb{e}^{n+1}\bar{\pmb{U}}^{n+1}\bigr)+\bigl( \bigl\vert \pmb{U}^{n} \bigr\vert ^{2}+ \bigl\vert \pmb{U} ^{n} \bigr\vert ^{2}\bigr) \bigl(\pmb{U}^{n}\bar{\pmb{e}}^{n}+ \pmb{e}^{n}\bar{\pmb{U}}^{n}\bigr) \\& \hphantom{\pmb{G}^{n}=}{}+ \bigl\vert \pmb{u}^{n} \bigr\vert ^{2}\bigl( \pmb{U}^{n+1}\bar{\pmb{e}}^{n+1}+\pmb{e}^{n+1}\bar{\pmb{u}} ^{n+1}\bigr)+ \bigl\vert \pmb{u}^{n+1} \bigr\vert ^{2}\bigl(\pmb{U}^{n}\bar{\pmb{e}}^{n}+ \pmb{e}^{n}\bar{\pmb{u}} ^{n}\bigr)\bigr]\bigl( \pmb{U}^{n+1}+\pmb{U}^{n}\bigr) \\& \hphantom{\pmb{G}^{n}=}{}+\bigl( \bigl\vert \pmb{u}^{n+1} \bigr\vert ^{4}+ \bigl\vert \pmb{u}^{n+1} \bigr\vert ^{2} \bigl\vert \pmb{u}^{n} \bigr\vert ^{2}+ \bigl\vert \pmb{u} ^{n} \bigr\vert ^{4}\bigr) \bigl(\pmb{e}^{n+1}+ \pmb{e}^{n}\bigr). \end{aligned}$$

Similarly, we obtain

4.13 $$\begin{aligned} &\bigl\Vert \pmb{G}^{n} \bigr\Vert ^{2}\leq C\bigl( \bigl\Vert \pmb{e}^{n} \bigr\Vert ^{2}+ \bigl\Vert \pmb{e}^{n+1} \bigr\Vert ^{2}\bigr), \end{aligned}$$

4.14 $$\begin{aligned} &\bigl\Vert \delta_{x}\pmb{G}^{n} \bigr\Vert ^{2}\leq C\bigl( \bigl\Vert \pmb{e}^{n} \bigr\Vert ^{2}+ \bigl\Vert \pmb{e} ^{n+1} \bigr\Vert ^{2}+ \bigl\Vert \delta_{x} \pmb{e}^{n} \bigr\Vert ^{2}+ \bigl\Vert \delta_{x} \pmb{e}^{n+1} \bigr\Vert ^{2}\bigr). \end{aligned}$$

Computing the inner product of (4.9) with $e^{n+1}+e^{n}$ and taking the imaginary part, we have

4.15 $$\begin{aligned} &\operatorname{Im}\bigl(\pmb{r}^{n}, \pmb{e}^{n}+\pmb{e}^{n+1}\bigr) \\ &\quad = \operatorname{Im}\bigl(i \delta_{t}\pmb{e}^{n},\pmb{e} ^{n}+ \pmb{e}^{n+1}\bigr)+\operatorname{Im}\bigl(\pmb{H}\delta^{2}_{x} \bigl(\pmb{e}^{n}+\pmb{e}^{n+1}\bigr),\pmb{e} ^{n}+ \pmb{e}^{n+1}\bigr) -\frac{1}{2}\operatorname{Im}\bigl(\pmb{F}^{n}, \pmb{e}^{n}+\pmb{e} ^{n+1}\bigr) \\ &\qquad {}-\frac{1}{3}\operatorname{Im}\bigl(\pmb{G}^{n},\pmb{e}^{n}+ \pmb{e}^{n+1}\bigr)-\frac{1}{2}\operatorname{Im}\bigl(\pmb{f} ^{n+\frac{1}{2}} \bigl(\pmb{e}^{n}+\pmb{e}^{n+1}\bigr),\pmb{e}^{n}+ \pmb{e}^{n+1}\bigr). \end{aligned}$$

For each term on the right-hand side of (4.15), we bound them as follows:

4.16 $$ \operatorname{Im}\bigl(i\delta_{t}\pmb{e}^{n},\pmb{e}^{n}+ \pmb{e}^{n+1}\bigr)=\frac{1}{\tau }\bigl( \bigl\Vert \pmb{e}^{n+1} \bigr\Vert ^{2}- \bigl\Vert \pmb{e}^{n} \bigr\Vert ^{2}\bigr). $$

As to the second term

4.17 $$ \operatorname{Im}\bigl(\pmb{H}\delta^{2}_{x}\bigl(\pmb{e}^{n}+ \pmb{e}^{n+1}\bigr),\pmb{e}^{n}+\pmb{e} ^{n+1}\bigr)=4 \operatorname{Im}\bigl(\pmb{R}\delta_{x}\pmb{e}^{n+\frac{1}{2}},\pmb{R}\delta _{x}\pmb{e}^{n+\frac{1}{2}}\bigr)=0. $$

For the last three terms on the right-hand side of (4.15), by using the Cauchy–Schwarz inequality, we obtain

4.18 $$\begin{aligned} & \biggl\vert \frac{1}{2}\operatorname{Im}\bigl(\pmb{F}^{n}, \pmb{e}^{n}+\pmb{e}^{n+1}\bigr) \biggr\vert \leq \frac{1}{4}\biggl( \bigl\Vert \pmb{F}^{n} \bigr\Vert ^{2}+\frac{1}{2}\bigl( \bigl\Vert \pmb{e}^{n} \bigr\Vert ^{2}+ \bigl\Vert \pmb{e} ^{n+1} \bigr\Vert ^{2}\bigr)\biggr) \\ &\hphantom{\biggl\vert \frac{1}{2}\operatorname{Im}\bigl(\pmb{F}^{n}, \pmb{e}^{n}+\pmb{e}^{n+1}\bigr) \biggr\vert }\leq C\bigl( \bigl\Vert \pmb{e}^{n} \bigr\Vert ^{2}+ \bigl\Vert \pmb{e}^{n+1} \bigr\Vert ^{2}\bigr), \end{aligned}$$

4.19 $$\begin{aligned} & \biggl\vert \frac{1}{3}\operatorname{Im}\bigl(\pmb{G}^{n}, \pmb{e}^{n}+\pmb{e}^{n+1}\bigr) \biggr\vert \leq \frac{1}{6}\biggl( \bigl\Vert \pmb{G}^{n} \bigr\Vert ^{2}+\frac{1}{2}\bigl( \bigl\Vert \pmb{e}^{n} \bigr\Vert ^{2}+ \bigl\Vert \pmb{e} ^{n+1} \bigr\Vert ^{2}\bigr)\biggr) \\ &\hphantom{\biggl\vert \frac{1}{3}\operatorname{Im}\bigl(\pmb{G}^{n}, \pmb{e}^{n}+\pmb{e}^{n+1}\bigr) \biggr\vert }\leq C\bigl( \bigl\Vert \pmb{e}^{n} \bigr\Vert ^{2}+ \bigl\Vert \pmb{e}^{n+1} \bigr\Vert ^{2}\bigr), \end{aligned}$$

4.20 $$\begin{aligned} &\frac{1}{2}\operatorname{Im}\bigl(\pmb{f}^{n+\frac{1}{2}}\bigl(\pmb{e}^{n}+ \pmb{e}^{n+1}\bigr),\pmb{e} ^{n}+\pmb{e}^{n+1}\bigr)\leq C\bigl( \bigl\Vert \pmb{e}^{n} \bigr\Vert ^{2}+ \bigl\Vert \pmb{e}^{n+1} \bigr\Vert ^{2}\bigr). \end{aligned}$$

For the term on the left-hand side of (4.15), we have

4.21 $$ \operatorname{Im}\bigl(\pmb{r}^{n},\pmb{e}^{n}+ \pmb{e}^{n+1}\bigr)\leq \frac{1}{2}\biggl( \bigl\Vert \pmb{r} ^{n} \bigr\Vert ^{2}+\frac{1}{2}\bigl( \bigl\Vert \pmb{e}^{n} \bigr\Vert ^{2}+ \bigl\Vert \pmb{e}^{n+1} \bigr\Vert ^{2}\bigr)\biggr). $$

From (4.15)–(4.21), we can obtain

4.22 $$ \bigl\Vert \pmb{e}^{n+1} \bigr\Vert ^{2}- \bigl\Vert \pmb{e}^{n} \bigr\Vert ^{2}\leq \tau \bigl( \bigl\Vert \pmb{r}^{n} \bigr\Vert ^{2}+C\tau \bigl( \bigl\Vert \pmb{e}^{n} \bigr\Vert ^{2}+ \bigl\Vert \pmb{e}^{n+1} \bigr\Vert ^{2}\bigr)\bigr). $$

Summing (4.22) up for *n*, we have

4.23 $$ \bigl\Vert \pmb{e}^{n} \bigr\Vert ^{2}\leq \bigl[O \bigl(h^{4}+\tau^{2}\bigr)\bigr]^{2}+C\tau \sum ^{n}_{l=1}\bigl( \bigl\Vert \pmb{e}^{l} \bigr\Vert ^{2}+ \bigl\Vert \pmb{e}^{l+1} \bigr\Vert ^{2}\bigr). $$

When *τ* is small enough, it follows from Lemma 4.2 that

4.24 $$ \bigl\Vert \pmb{e}^{n} \bigr\Vert \leq O \bigl(h^{4}+\tau^{2}\bigr). $$

Computing the inner product of (4.8) with $\delta_{t}\pmb{e} ^{n}$ and taking the real part, we have

4.25 $$\begin{aligned} \operatorname{Re}\bigl(\pmb{r}^{n}, \delta_{t}\pmb{e}^{n}\bigr)= {}&\operatorname{Re}\bigl(i \delta_{t}\pmb{e}^{n}, \delta_{t} \pmb{e}^{n}\bigr)+\frac{1}{2}\operatorname{Re}\bigl(\pmb{H} \delta^{2}_{x}\bigl(\pmb{e} ^{n}+ \pmb{e}^{n+1}\bigr),\delta_{t}\pmb{e}^{n}\bigr) - \frac{1}{4}\operatorname{Re}\bigl(\pmb{F}^{n}, \delta_{t} \pmb{e}^{n}\bigr) \\ &{}-\frac{1}{6}\operatorname{Re}\bigl(\pmb{G}^{n},\delta_{t} \pmb{e}^{n}\bigr)-\frac{1}{2}\operatorname{Re}\bigl(\pmb{f} ^{n+\frac{1}{2}} \bigl(\pmb{e}^{n}+\pmb{e}^{n+1}\bigr),\delta_{t} \pmb{e}^{n}\bigr). \end{aligned}$$

For each term on the right-hand side of (4.25), we bound them as follows:

4.26 $$ \operatorname{Re}\bigl(i\delta_{t}\pmb{e}^{n},\delta_{t} \pmb{e}^{n}\bigr)=0. $$

For the second term, it follows from Lemma 3.1 that

4.27 $$ \frac{1}{2}\operatorname{Re}\bigl(\pmb{H}\delta^{2}_{x}\bigl( \pmb{e}^{n}+\pmb{e}^{n+1}\bigr),\delta _{t} \pmb{e}^{n}\bigr)=\frac{-1}{2\tau }\bigl( \bigl\Vert \pmb{R} \delta_{x}\pmb{e}^{n+1} \bigr\Vert ^{2}- \bigl\Vert \pmb{R}\delta_{x}\pmb{e}^{n} \bigr\Vert ^{2}\bigr). $$

As to the third term, it follows from (4.8) that

4.28 $$\begin{aligned} \begin{aligned} \delta_{t}\pmb{e}^{n}=i \frac{1}{2}\pmb{H}\delta^{2}_{x}\bigl( \pmb{e}^{n+1}+\pmb{e} ^{n}\bigr) -\frac{i}{4} \pmb{F}^{n}-\frac{i}{6}\pmb{G}^{n} -i \pmb{f}^{n+ \frac{1}{2}}\frac{\pmb{e}^{n+1}+\pmb{e}^{n}}{2}-i\pmb{r}^{n}. \end{aligned} \end{aligned}$$

By using the Cauchy–Schwarz inequality, we obtain

4.29 $$\begin{aligned} \operatorname{Re}\bigl(\pmb{F}^{n},\delta_{t} \pmb{e}^{n}\bigr)= {}&\frac{1}{2}\operatorname{Re}\biggl(\pmb{F}^{n},i \pmb{H} \delta^{2}_{x}\bigl(\pmb{e}^{n+1}+ \pmb{e}^{n}\bigr) -\frac{i}{4}\pmb{F}^{n}- \frac{i}{6}\pmb{G}^{n}-i\pmb{f}^{n+\frac{1}{2}}\frac{\pmb{e}^{n+1}+\pmb{e} ^{n}}{2}-i \pmb{r}^{n}\biggr) \\ = {}&\frac{-1}{2}\operatorname{Im}\bigl(\pmb{F}^{n},\pmb{H} \delta^{2}_{x}\bigl(\pmb{e}^{n+1}+\pmb{e} ^{n}\bigr)\bigr)+\frac{1}{6}\operatorname{Im}\bigl(\pmb{F}^{n}, \pmb{G}^{n}\bigr) \\ &{}+\operatorname{Im}\biggl(\pmb{F}^{n},\pmb{f}^{n+\frac{1}{2}}\frac{\pmb{e}^{n+1}+\pmb{e} ^{n}}{2} \biggr)+\operatorname{Im}\bigl(\pmb{F}^{n},\pmb{r}^{n}\bigr) \\ = {}&\frac{1}{2}\operatorname{Im}\bigl(\pmb{R}\delta_{x}\pmb{F}^{n}, \pmb{R}\delta_{x}\bigl(\pmb{e} ^{n+1}+\pmb{e}^{n} \bigr)\bigr)+\frac{1}{6}\operatorname{Im}\bigl(\pmb{F}^{n}, \pmb{G}^{n}\bigr) \\ &{}+\operatorname{Im}\biggl(\pmb{F}^{n},\pmb{f}^{n+\frac{1}{2}}\frac{\pmb{e}^{n+1}+\pmb{e} ^{n}}{2} \biggr)+\operatorname{Im}\bigl(\pmb{F}^{n},\pmb{r}^{n}\bigr), \end{aligned}$$

where

$$\begin{aligned} &\operatorname{Im}\bigl(\pmb{R}\delta_{x} \pmb{F}^{n},\pmb{R}\delta_{x}\bigl(\pmb{e}^{n+1}+ \pmb{e} ^{n}\bigr)\bigr) \\ &\quad \leq \frac{1}{2}\biggl( \bigl\Vert \pmb{R}\delta_{x}\pmb{F}^{n} \bigr\Vert ^{2}+ \frac{1}{2}\bigl( \bigl\Vert \pmb{R}\delta_{x} \pmb{e}^{n+1} \bigr\Vert ^{2}+ \bigl\Vert \pmb{R}\delta _{x}\pmb{e}^{n} \bigr\Vert ^{2}\bigr)\biggr) \\ &\quad \leq C_{1}\bigl( \bigl\Vert \delta_{x} \pmb{F}^{n} \bigr\Vert ^{2}+ \bigl\Vert \delta_{x}\pmb{e}^{n+1} \bigr\Vert ^{2}+ \bigl\Vert \delta_{x}\pmb{e}^{n} \bigr\Vert ^{2} \bigr) \\ &\quad \leq C\bigl( \bigl\Vert \pmb{e}^{n+1} \bigr\Vert ^{2}+ \bigl\Vert \pmb{e}^{n} \bigr\Vert ^{2}+ \bigl\Vert \pmb{R}\delta _{x}\pmb{e}^{n+1} \bigr\Vert ^{2}+ \bigl\Vert \pmb{R}\delta_{x}\pmb{e}^{n} \bigr\Vert ^{2}\bigr), \\ &\operatorname{Im}\biggl(\pmb{F}^{n},\pmb{f}^{n+\frac{1}{2}}\frac{\pmb{e}^{n+1}+\pmb{e} ^{n}}{2}\biggr) \\ &\quad \leq \frac{1}{2} \biggl( \bigl\Vert \pmb{F}^{n} \bigr\Vert ^{2}+\frac{M_{1}}{2}\bigl( \bigl\Vert \pmb{e} ^{n+1} \bigr\Vert ^{2}+ \bigl\Vert \pmb{e}^{n} \bigr\Vert ^{2}\bigr)\biggr) \\ &\quad \leq C\bigl( \bigl\Vert \pmb{e}^{n+1} \bigr\Vert ^{2}+ \bigl\Vert \pmb{e}^{n} \bigr\Vert ^{2}\bigr), \\ &\operatorname{Im}\bigl(\pmb{F}^{n},\pmb{r}^{n}\bigr)\leq \frac{1}{2}\bigl( \bigl\Vert \pmb{F}^{n} \bigr\Vert ^{2}+ \bigl\Vert \pmb{r} ^{n} \bigr\Vert ^{2} \bigr)\leq C\bigl( \bigl\Vert \pmb{e}^{n+1} \bigr\Vert ^{2}+ \bigl\Vert \pmb{e}^{n} \bigr\Vert ^{2}+ \bigl\Vert \pmb{r} ^{n} \bigr\Vert ^{2}\bigr), \\ &\operatorname{Im}\bigl(\pmb{F}^{n},\pmb{G}^{n}\bigr)\leq \frac{1}{2}\bigl( \bigl\Vert \pmb{F}^{n} \bigr\Vert ^{2}+ \bigl\Vert \pmb{G} ^{n} \bigr\Vert ^{2} \bigr)\leq C\bigl( \bigl\Vert \pmb{e}^{n+1} \bigr\Vert ^{2}+ \bigl\Vert \pmb{e}^{n} \bigr\Vert ^{2}\bigr). \end{aligned}$$

Then we have

4.30 $$ \operatorname{Re}\bigl(\pmb{F}^{n},\delta_{t}\pmb{e}^{n}\bigr) \leq C\bigl( \bigl\Vert \pmb{e}^{n+1} \bigr\Vert ^{2}+ \bigl\Vert \pmb{e} ^{n} \bigr\Vert ^{2}+ \bigl\Vert \pmb{r}^{n} \bigr\Vert ^{2}+ \bigl\Vert \pmb{R} \delta_{x}\pmb{e}^{n+1} \bigr\Vert ^{2}+ \bigl\Vert \pmb{R}\delta_{x}\pmb{e}^{n} \bigr\Vert ^{2}\bigr). $$

Similarly, we obtain

4.31 $$\begin{aligned} &\operatorname{Re}\bigl(\pmb{G}^{n},\delta_{t}\pmb{e}^{n} \bigr)\leq C\bigl( \bigl\Vert \pmb{e}^{n+1} \bigr\Vert ^{2}+ \bigl\Vert \pmb{e} ^{n} \bigr\Vert ^{2}+ \bigl\Vert \pmb{r}^{n} \bigr\Vert ^{2}+ \bigl\Vert \pmb{R} \delta_{x}\pmb{e}^{n+1} \bigr\Vert ^{2}+ \bigl\Vert \pmb{R}\delta_{x}\pmb{e}^{n} \bigr\Vert ^{2}\bigr), \end{aligned}$$

4.32 $$\begin{aligned} &\operatorname{Re}\biggl(\pmb{f}^{n+\frac{1}{2}}\frac{\pmb{e}^{n+1}+\pmb{e}^{n}}{2},\delta _{t} \pmb{e}^{n}\biggr) \\ &\quad \leq C\bigl( \bigl\Vert \pmb{e}^{n+1} \bigr\Vert ^{2}+ \bigl\Vert \pmb{e}^{n} \bigr\Vert ^{2}+ \bigl\Vert \pmb{r} ^{n} \bigr\Vert ^{2}+ \bigl\Vert \pmb{R}\delta_{x}\pmb{e}^{n+1} \bigr\Vert ^{2}+ \bigl\Vert \pmb{R}\delta_{x}\pmb{e} ^{n} \bigr\Vert ^{2}\bigr). \end{aligned}$$

From (4.25)–(4.32), we can obtain

4.33 $$\begin{aligned}& \bigl\Vert \pmb{R}\delta_{x}\pmb{e}^{n+1} \bigr\Vert ^{2}- \bigl\Vert \pmb{R}\delta_{x} \pmb{e}^{n} \bigr\Vert ^{2} \\& \quad \leq \tau C\tau \bigl( \bigl\Vert \pmb{R}\delta_{x}\pmb{e}^{n} \bigr\Vert ^{2}+ \bigl\Vert \pmb{R} \delta_{x}\pmb{e}^{n+1} \bigr\Vert ^{2}\bigr))+\operatorname{Re}\bigl(\pmb{r}^{n}, \delta_{t}\pmb{e}^{n}\bigr)+ \tau \bigl[O \bigl(h^{4}+\tau^{2}\bigr)\bigr]^{2}. \end{aligned}$$

Summing (4.33) up for *n*, we obtain

4.34 $$\begin{aligned} \bigl\Vert \pmb{e}^{n} \bigr\Vert ^{2} \leq& C\tau \bigl[O\bigl(h^{4}+\tau^{2}\bigr) \bigr]^{2}+C\tau \sum^{n} _{l=1} \bigl( \bigl\Vert \pmb{R}\delta_{x}\pmb{e}^{l} \bigr\Vert ^{2}+ \bigl\Vert \pmb{R}\delta_{x}\pmb{e} ^{l+1} \bigr\Vert ^{2}\bigr)) \\ &{} +C\tau \sum ^{n}_{l=1}\operatorname{Re}\bigl(\pmb{r}^{l}, \delta_{t}\pmb{e} ^{l}\bigr). \end{aligned}$$

From Lemma 4.4 and Lemma 4.5, we have

4.35 $$ \Biggl\vert \tau \operatorname{Re}\sum^{n}_{l=1} \operatorname{Re}\bigl(\pmb{r}^{l},\delta_{t}\pmb{e}^{l}\bigr) \Biggr\vert \leq \bigl[O\bigl(h ^{4}+\tau^{2}\bigr) \bigr]^{2}. $$

Substituting (4.35) into (4.34) and applying the discrete Gronwall inequality when taking *τ* sufficiently small, we have

4.36 $$ \bigl\Vert \pmb{R}\delta_{x}\pmb{e}^{n} \bigr\Vert \leq \bigl[O\bigl(h^{4}+\tau^{2}\bigr)\bigr]. $$

Then, from Lemma 4.3, we have

4.37 $$ \bigl\Vert \delta_{x}\pmb{e}^{n} \bigr\Vert \leq \bigl[O\bigl(h^{4}+\tau^{2}\bigr)\bigr]. $$

From (4.2) and (4.34), and using Lemma 4.1, we have

4.38 $$ \bigl\Vert \pmb{e}^{n} \bigr\Vert _{\infty }\leq \bigl[O \bigl(h^{4}+\tau^{2}\bigr)\bigr]. $$

 □

Similarly, we can prove the stability of the difference solution.

### Theorem 4.3

*Under the conditions of Theorem* 4.2, *the solution of the difference scheme* (2.2)*–*(2.4) *is unconditionally stable for initial data in the* $\|\cdot \|_{\infty }$ *norm*.

## Numerical experiment

In this section, we consider the following problem:

5.1 $$\begin{aligned} &i\frac{\partial u}{\partial t}+\frac{\partial^{2} u}{\partial x^{2}}-\bigl( \vert u \vert ^{2}+ \vert u \vert ^{4}\bigr)u \\ &\quad =\bigl[4(x-2t)^{2}-e^{-2(x-2t)^{2}}-e ^{-4(x-2t)^{2}}\bigr]u\quad (-15 < x < 15,0< t \leq 1), \end{aligned}$$

5.2 $$\begin{aligned} &u(x, 0)=e^{-x^{2}+ix}\quad (-15 < x < 15), \end{aligned}$$

5.3 $$\begin{aligned} &u(-15, t)=u(15, t)=0\quad (0 < t \leq 1). \end{aligned}$$

An exact solution is given by

5.4 $$ u(x,t)=e^{-(x-2t)^{2}+i(x-3t)}. $$

For problems (5.1)–(5.3), we have the following CFD scheme:

5.5 $$\begin{aligned} &\frac{i}{\tau }A_{h} \bigl(u^{n+1}_{j}-u^{n}_{j}\bigr)+ \frac{1}{2}\delta^{2} _{x}\bigl(u^{n+1}_{j}+u^{n}_{j} \bigr) -\frac{1}{4}A_{h}\bigl[\bigl( \bigl\vert u^{n+1}_{j} \bigr\vert ^{2}+ \bigl\vert u ^{n}_{j} \bigr\vert ^{2}\bigr) \bigl(u^{n+1}_{j}+u^{n}_{j}\bigr)\bigr] \\ &\quad {}-\frac{1}{6}A_{h}\bigl[\bigl( \bigl\vert u^{n+1}_{j} \bigr\vert ^{4}+ \bigl\vert u^{n+1}_{j} \bigr\vert ^{2} \bigl\vert u^{n}_{j} \bigr\vert ^{2}+ \bigl\vert u ^{n}_{j} \bigr\vert ^{4}\bigr) \bigl(u^{n+1}_{j}+u^{n}_{j}\bigr) \bigr]=A_{h}\biggl[f^{n+\frac{1}{2}}\frac{u ^{n+1}_{j}+u^{n}_{j}}{2}\biggr] \\ &\quad (j=1, 2, \ldots, J-1, n=1, 2, \ldots, N-1), \end{aligned}$$

5.6 $$\begin{aligned} &u^{n}_{0}=u^{n}_{J}=0\quad (n=1, 2, \ldots, N), \end{aligned}$$

5.7 $$\begin{aligned} &u^{0}_{J}=u_{0}(x_{j})\quad (j=1, 2, \ldots, J). \end{aligned}$$

In order to obtain the numerical solution $u^{n+1}_{j}$, an iterative algorithm can be used. We define the following iterative algorithm:

5.8 $$ A^{n+1(s)}_{j-1}u^{n+1(s+1)}_{j-1}+ B^{n+1(s)}_{j}u^{n+1(s+1)}_{j}+ C ^{n+1(s)}_{j+1}u^{n+1(s+1)}_{j+1}= D^{n+1(s)}_{j}, $$

where *s* denotes the number of iteration, and

$$\begin{aligned} &A^{n+1(s)}_{j-1}=\frac{i}{12}+\frac{r}{2}- \frac{\tau }{48}E^{n+1(s)} _{j-1}-\frac{\tau }{72}F^{n+1(s)}_{j-1}- \frac{\tau }{24}f^{n+ \frac{1}{2}}_{j-1}, \\ &C^{n+1(s)}_{j+1}=\frac{i}{12}+\frac{r}{2}- \frac{\tau }{48}E^{n+1(s)} _{j+1}-\frac{\tau }{72}F^{n+1(s)}_{j+1}- \frac{\tau }{24}f^{n+ \frac{1}{2}}_{j+1}, \\ &B^{n+1(s)}_{j}=\frac{5i}{6}-r-\frac{5\tau }{24}E^{n+1(s)}_{j}- \frac{5 \tau }{36}F^{n+1(s)}_{j}-\frac{5\tau }{12}f^{n+\frac{1}{2}}_{j}, \\ &D^{n+1(s)}_{j}= \biggl( \frac{i}{12}-\frac{r}{2}+\frac{\tau }{48}E^{n+1(s)} _{j-1}+\frac{\tau }{72}F^{n+1(s)}_{j-1}+ \frac{\tau }{24}f^{n+ \frac{1}{2}}_{j-1}\biggr)u^{n}_{j-1} \\ &\hphantom{D^{n+1(s)}_{j}=}{}+\biggl(\frac{5i}{6}+r+\frac{5\tau }{24}E^{n+1(s)}_{j}+ \frac{5\tau }{36}F ^{n+1(s)}_{j}+\frac{5\tau }{12}f^{n+\frac{1}{2}}_{j} \biggr)u^{n}_{j} \\ &\hphantom{D^{n+1(s)}_{j}=}{}+\biggl(\frac{i}{12}-\frac{r}{2}+\frac{\tau }{48}E^{n+1(s)}_{j+1}+ \frac{ \tau }{72}F^{n+1(s)}_{j+1}+\frac{\tau }{24}f^{n+\frac{1}{2}}_{j+1} \biggr)u ^{n}_{j+1}. \end{aligned}$$

The initial value of iteration $u^{n+1(0)}_{j}=u^{n}_{j}$, when $\|u^{n+1(s+1)}-u^{n+1(s)}\|_{\infty }\leq \varepsilon $, it is the end of iteration (this paper has $\varepsilon =10^{-6}$).

In order to compare the efficiency of CFD scheme with reference to the scheme in [27, 28], we give their schemes

$$\begin{aligned} &i\delta_{\hat{t}}u^{n}_{j}+ \frac{1}{2}\delta^{2}_{x}\bigl(u^{n+1}_{j}+u ^{n-1}_{j}\bigr) -\frac{1}{2} \bigl\vert u^{n}_{j} \bigr\vert ^{2}\bigl(u^{n+1}_{j}+u^{n-1}_{j} \bigr) \\ &\qquad {}- \frac{1}{6}\bigl[\bigl( \bigl\vert u^{n+1}_{j} \bigr\vert ^{4}+ \bigl\vert u^{n+1}_{j} \bigr\vert ^{2} \bigl\vert u^{n-1}_{j} \bigr\vert ^{2}+ \bigl\vert u ^{n-1}_{j} \bigr\vert ^{4}\bigr) \bigl(u^{n+1}_{j}+u^{n-1}_{j} \bigr)\bigr] \\ &\quad =\biggl[f^{n}\frac{u^{n+1}_{j}+u^{n-1}_{j}}{2}\biggr]\quad (j=1, 2, \ldots, J-1, n=1, 2, \ldots, N-1), \\ &i\delta_{t}u^{n}_{j}+ \frac{1}{24}\bigl[\bigl(-\delta^{2}_{x}u^{n+1}_{j-1}+14 \delta^{2}_{x}u^{n+1}_{j}- \delta^{2}_{x}u^{n+1}_{j+1}\bigr)+\bigl(- \delta^{2} _{x}u^{n}_{j-1}+14 \delta^{2}_{x}u^{n}_{j}- \delta^{2}_{x}u^{n}_{j+1}\bigr)\bigr] \\ &\qquad {} - \frac{1}{4}\bigl( \bigl\vert u^{n+1}_{j} \bigr\vert ^{2}+ \bigl\vert u^{n}_{j} \bigr\vert ^{2}\bigr) \bigl(u^{n+1}_{j}+u^{n}_{j}\bigr)- \frac{1}{6}\bigl[\bigl( \bigl\vert u^{n+1}_{j} \bigr\vert ^{4}+ \bigl\vert u^{n+1}_{j} \bigr\vert ^{2} \bigl\vert u ^{n}_{j} \bigr\vert ^{2}+ \bigl\vert u^{n}_{j} \bigr\vert ^{4}\bigr) \bigl(u^{n+1}_{j}+u^{n}_{j} \bigr)\bigr] \\ &\quad =\biggl[f^{n+ \frac{1}{2}}\frac{u^{n+1}_{j}+u^{n}_{j}}{2}\biggr], \\ &\quad (j=1, 2, \ldots, J-1, n=1, 2, \ldots, N-1). \end{aligned}$$

For convenience, we denote the one in [27] as Scheme 2, and the one in [28] as Scheme 3, respectively.

**Figure 1:**
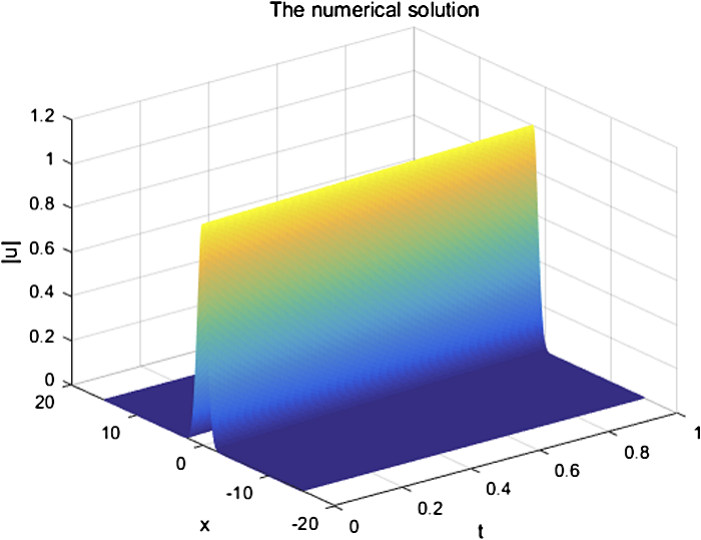
The numerical solution with $h=0.05$, $\tau = 0.0025$

**Figure 2:**
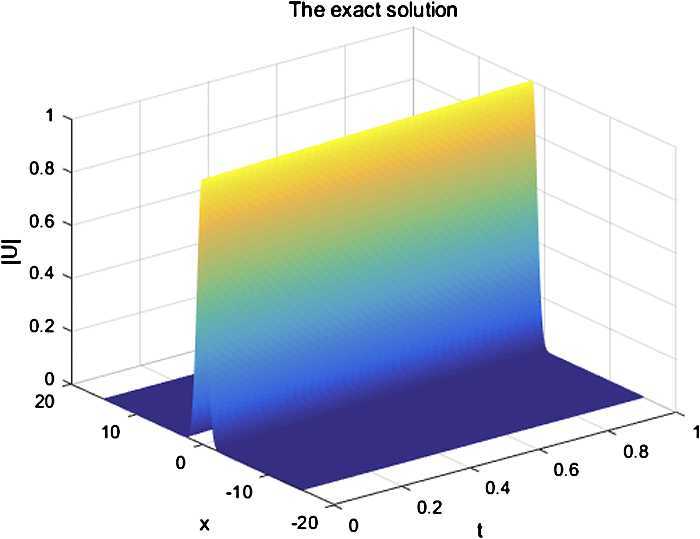
The exact solution with $h=0.05$, $\tau = 0.0025$

**Figure 3:**
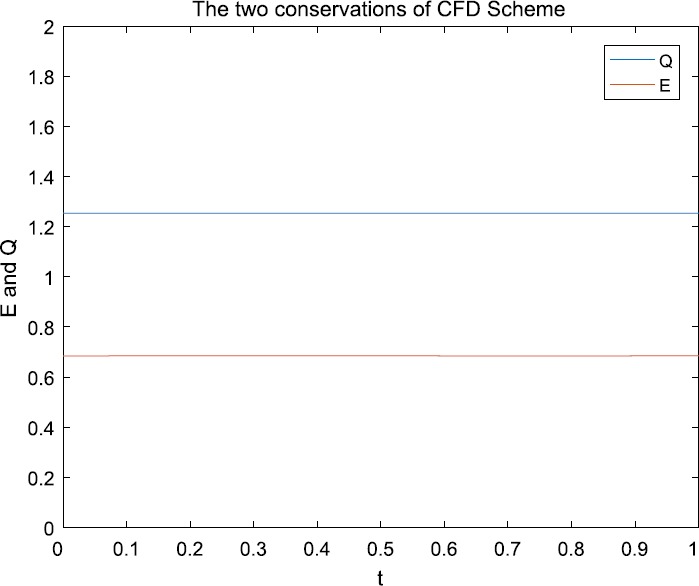
Discrete mass Q and energy E with $h= 0.1$, $\tau =0.01$

From Fig. 1 and Fig. 2, we can see that the numerical solution of the compact scheme and the exact solution are in good agreement. As shown in Table 1, the accuracy of CFD Scheme is higher than that of the other schemes. As indicated in Table 2, the CPU time of CFD Scheme has the same CPU time cost as that of Scheme 2 and Scheme 3 in computation. From Table 3, it is obvious that CFD Scheme is convergent in maximum norm, and the convergence order is $O(h^{4}+\tau^{2})$. Figure 3 indicates that the two conservations of CFD Scheme are very good.

**Table 1:** Comparison of the accuracy for the numerical solutions

*t*	CFD Scheme	Scheme 2	Scheme 3
0.2	3.1712e-4	4.8000e-3	3.5170e-4
0.4	6.0713e-4	8.2000e-3	6.5370e-4
0.6	7.9096e-4	1.1300e-2	8.5260e-4
0.8	8.4966e-4	1.2700e-2	9.1910e-4
1.0	7.7363e-4	1.1400e-2	8.2220e-4

**Table 2:** CPU time of the three schemes

*h*	*t*	CFD Scheme	Scheme 2	Scheme 3
0.2	0.04	0.80 s	0.81 s	0.58 s
0.1	0.01	5.83 s	6.49 s	6.23 s
0.05	0.0025	62.21 s	64.05 s	40.79 s

**Table 3:** Errors and convergence order at difference steps

*h*	*τ*	$E_{\infty }(h, \tau)$	$E_{\infty }(2h, 4\tau)/E_{\infty }(h, \tau)$
0.2	0.04	1.3693e-2	
0.1	0.01	8.4966e-4	16.12
0.05	0.0025	5.3247e-5	15.89

## Conclusion

In this paper, a compact finite difference scheme is constructed for the nonlinear Schrödinger equation involving a quintic term. The discrete maximum norm error estimates show that the proposed schemes are in second and fourth order accurate in time and space, respectively. In numerical experiment, numerical results are carried out to confirm the theoretical analysis.
